# Application of multiple sgRNAs boosts efficiency of CRISPR/Cas9-mediated gene targeting in Arabidopsis

**DOI:** 10.1186/s12915-024-01810-7

**Published:** 2024-01-17

**Authors:** Jing Li, Dali Kong, Yongping Ke, Wenjie Zeng, Daisuke Miki

**Affiliations:** 1 Shanghai Center for Plant Stress Biology, CAS Center for Excellence in Molecular Plant Sciences, Chinese Academy of Sciences, Shanghai, 200032 China; 2 University of Chinese Academy of Sciences, Beijing, 100049 China

**Keywords:** Genome engineering, Gene targeting, CRISPR/Cas9, Multiple sgRNAs, Arabidopsis

## Abstract

**Background:**

Precise gene targeting (GT) is a powerful tool for heritable precision genome engineering, enabling knock-in or replacement of the endogenous sequence via homologous recombination. We recently established a CRISPR/Cas9-mediated approach for heritable GT in *Arabidopsis thaliana* (Arabidopsis) and rice and reported that the double-strand breaks (DSBs) frequency of Cas9 influences the GT efficiency. However, the relationship between DSBs and GT at the same locus was not examined. Furthermore, it has never been investigated whether an increase in the number of copies of sgRNAs or the use of multiple sgRNAs would improve the efficiency of GT.

**Results:**

Here, we achieved precise GT at endogenous loci *Embryo Defective 2410* (*EMB2410*) and *Repressor of Silencing 1* (*ROS1*) using the sequential transformation strategy and the combination of sgRNAs. We show that increasing of sgRNAs copy number elevates both DSBs and GT efficiency. On the other hand, application of multiple sgRNAs does not always enhance GT efficiency. Our results also suggested that some inefficient sgRNAs would play a role as a helper to facilitate other sgRNAs DSBs activity.

**Conclusions:**

The results of this study clearly show that DSB efficiency, rather than mutation pattern, is one of the most important key factors determining GT efficiency. This study provides new insights into the relationship between sgRNAs, DSBs, and GTs and the molecular mechanisms of CRISPR/Cas9-mediated GTs in plants.

**Supplementary Information:**

The online version contains supplementary material available at 10.1186/s12915-024-01810-7.

## Background

Precise genomic sequence modification through genome engineering must be a powerful tool for many biotechnological applications in research and molecular breeding [1]. Genome editing technology has made great advances in plant biotechnology in recent decades, enabling the creation of genome-edited crops for biomass and food production [2]. Engineered sequence-specific nucleases (SSNs) that generate double-strand breaks (DSBs) at target DNA sites in a sequence-specific manner have provided the basis for gene editing in plants. The mechanism of DSB repair in eukaryotes is highly conserved, with DSBs being repaired predominantly by the non-homologous end-joining (NHEJ) pathway and rarely by the homology-directed repair (HDR) pathway. The error-prone NHEJ pathway is the primary repair mechanism for site-specific DSBs induced by SSNs, causing random mutations such as short in-del. The HDR pathway, on the other hand, causes error-free and precise knock-ins (KIs) and sequence substitutions when a specific repair template with homology arms of a certain length is provided [3].

HDR-mediated gene targeting (GT) is a useful technology for introducing desired sequences or replacing genomic sequences. However, the low frequency of HDRs, especially in seed plants, is an obstacle to efficient plant genome engineering. Possible reasons for the low efficiency of GT in plants are that HDR activity is specific to the S to late G2 phase of the cell cycle and the difficulty of delivering the donor template due to the cell wall [4, 5]. Therefore, many studies are contributing to improving the efficiency of HDR-mediated GT in plants. For example, the frequency of HDR can be increased by regulating the expression of factors involved in these two major repair pathways, NHEJ and HDR [1, 6–8]; using donor templates to associate with target sites and regulating the cell cycle to improve the spatiotemporal availability of donors [9–13], using the viral replicon or the biolistic method to increase the dose of donor template [14–17], and optimizing the type or structure of donor template required for HDR [18–21].

The clustered regularly interspaced short palindromic repeat (CRISPR)/CRISPR-associated protein 9 (CRISPR/Cas9) system has dominated the field of genome editing over the past decade and has shown great potential in plant functional genomics and crop improvement. The *Streptococcus pyogenes* Cas9 (SpCas9; hereafter Cas9) system has two major components: the Cas9 protein and single guide RNA (sgRNA). The functional complex of Cas9 and sgRNA recognizes complementary sequences at target loci with protospacer adjacent motifs (PAMs) and induces DSBs. The frequency of DSBs by the CRISPR/Cas9 system is thought to be highly dependent on the primary DNA sequence of the target sequence [22, 23]. Therefore, online predictive web resources are being used to select sgRNAs with high specificity and efficiency for mutagenesis and GT [24]. Further studies have revealed that the secondary structure of sgRNAs affects Cas9 targeting efficiency [25–27]. Hence, the design of sgRNAs is crucial to improve GT efficiency and should reflect the chromosomal environment of the target site as well as its nucleotide sequence [28–30].

We reported a sequential transformation strategy that improves the efficiency and precision of GT in *Arabidopsis thaliana* (Arabidopsis) and rice [12, 31]. Specifically, the plant with Cas9 protein expression background was used as the parental line, and secondary transformation was performed by using donor plasmids for efficient GT. Our results showed that GT efficiency depends on sgRNA design and DSBs frequency [32]. However, the previous study was conducted at different loci and the relationship between DSB and GT at the same locus has not been examined. Here, we examined the relationship between the four sgRNAs and GT efficiency at two different endogenous loci. The results showed a statistically clear positive correlation between DSB frequency and GT efficiency. This indicates that if the sgRNA is not properly designed, it is difficult to obtain the desired GT. To overcome the problem of low GT efficiency caused by inefficient Cas9, targeting the gene with two different sgRNAs was shown to generate more DSBs, activate more HDRs, and improve the KI efficiency of swine herpesviruses [33]. Furthermore, it has been reported that in plants, sgRNA expression levels and the use of multiple sgRNAs are both critical for DSBs frequency [34, 35]. In addition, studies with mouse cell lines revealed that deletion of short sequences near the target site may favor the HDR process using overlapping sgRNAs compared to non-overlapping sgRNAs causing longer deletions [36, 37]. However, no study has applied multiple sgRNAs in plants to analyze the GT efficiency of a single target locus. Therefore, this study intends to increase the frequency of DSBs and improve GT efficiency by using multiple sgRNAs. As a result, multiple sgRNAs strategy can increase the frequency of DSBs, but not always improve the GT efficiency. On the other hand, the multiple-sgRNA strategy compensates for insufficiently active sgRNAs at the same target locus, thereby alleviating the low GT efficiency caused by the single sgRNA.

## Results

### Design and evaluation of sgRNA activities

**Fig. 1:**
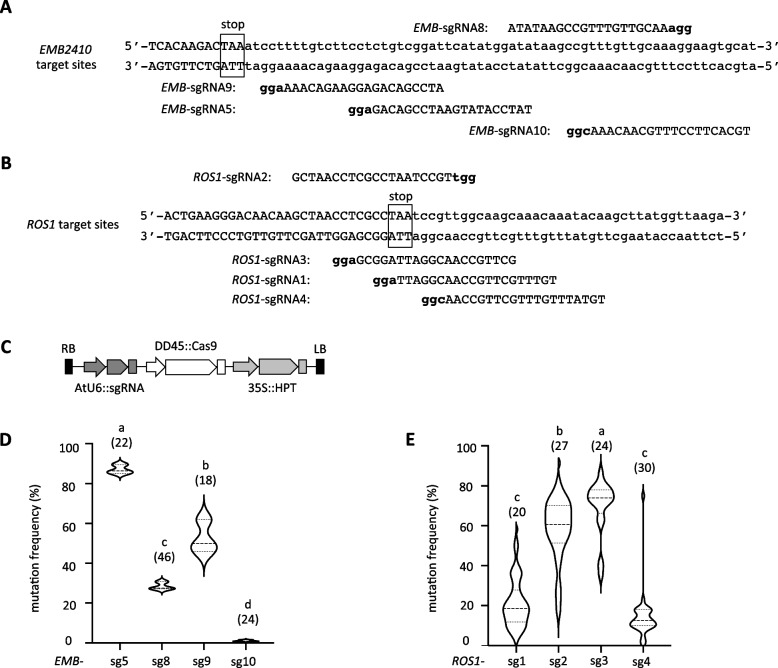
Evaluation of sgRNAs activity at two target loci in Arabidopsis. **A**, **B** Design of sgRNAs targeting the *EMB2410* (**A**) and *ROS1* (**B**) loci, respectively. Lowercase and boldface letters in the sgRNA sequence indicate PAM sequences. The coding sequence is shown in uppercase, the 3′ UTR in lowercase, and the stop codon in square to the target site sequence. *EMB*-sgRNA5 and *ROS1*-sgRNA2 have been used in previous studies. **C** Schematic diagram of CRISPR/Cas9 system construct. The construct consists of one AtU6 promoter-driven sgRNA expression cassette and a DD45 promoter-driven SpCas9 expression cassette. T1 transformants were selected for hygromycin resistance of 35S::HPT. **D**, **E** Evaluation of sgRNA activity in T1 transformants targeting the *EMB2410* (**D**) and *ROS1* (**E**) loci, respectively. The numbers in parenthesis represent the number of individual samples analyzed. One way ANOVA and Tukey test were used for difference analysis between data (*P* < 0.05)

We have reported efficient and precise GT events at wide range of endogenous loci in Arabidopsis. Firstly, we aimed to investigate the relationship between DSB frequency and GT efficiency using single sgRNA at the selected two target genes, *Embryo Defective 2410* (*EMB2410*) and *Repressor of Silencing 1* (*ROS1*), which have high GT efficiency and to which the same donor constructs from previous studies can be applied [32]. The GT efficiency of the sequential transformation method is higher than that of the all-in-one method [12]. Nevertheless, our previous studies have shown that not all target genes are successful in GT [32]. Undeniably, many unknown factors are involved in GT, but we have reasonable grounds to speculate that improperly designed sgRNAs are responsible for this outcome [32]. Therefore, our objective is to examine the correlation between DSB and GT for individual genes using a variety of sgRNAs. Using the online tool CRISPOR, four sgRNAs were designed for each target site of the two genes and named *EMB*-sgRNA5, *EMB*-sgRNA8, *EMB*-sgRNA9, *EMB*-sgRNA10, *ROS1*-sgRNA1, *ROS1*-sgRNA2, *ROS1*-sgRNA3, and *ROS1*-sgRNA4 (Fig. 1A, B, Additional file 2: Table S1). These sgRNAs were designed at endogenous target sites, the region between the 5′ and 3′ homology arms that were designed in the previous study [32] (Fig. 1A, B, Additional file 1: FigS1), and selected according to their prediction scores by CRISPOR (Additional file 2: Table S1, S2). The two of these sgRNAs, *EMB*-sgRNA5 and *ROS1*-sgRNA2, were used in epitope-tag knock-in (KI) experiments in our previous studies [12, 32, 38]. The AtU6-26 promoter driven sgRNA cassette was cloned into the DD45 pro::Cas9 plasmid (Fig. 1C) and transformed into Arabidopsis Col-0 accession.

To evaluate the DSBs frequency induced by different sgRNAs, mutation frequencies were measured in 18 to 46 independent T1 transgenic plants for each construct. The results show significant differences in the activity of each sgRNA (Fig. 1D, E). At the *EMB2410* locus, *EMB*-sgRNA5 had the highest mutation frequency, followed by *EMB*-sgRNA9 and *EMB*-sgRNA8. In contrast, no clear DSB activity was observed for *EMB*-sgRNA10 (Fig. 1D). Analysis of mutation frequencies at the *ROS1* locus showed that *ROS1*-sgRNA3 had the highest efficiency, followed by *ROS1*-sgRNA2, *ROS1*-sgRNA1, and *ROS1*-sgRNA4 (Fig. 1E). The variation in capacity and ranking of the mutation frequencies of these sgRNAs closely matched the website predictions, but the actual mutation values did not perfectly match the predicted frequencies (Additional file 2: Table S1).

### GT efficiency correlates with DSB activity of single sgRNA

**Fig. 2:**
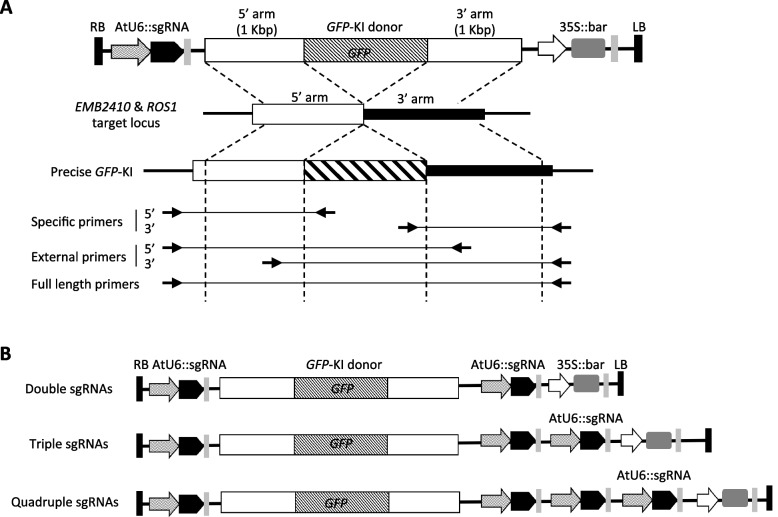
Multiple-sgRNA strategy for *GFP* knock-in. **A** Diagram of *GFP*-KI donor construct and primers for screening. The single sgRNA donor construct consists of one AtU6 promoter-driven sgRNA expression cassette and a KI *GFP* sequence with homologous arms of the endogenous target gene. Specific primers are specific for the GT allele, while external and full-length primers can amplify both endogenous and KI GT alleles. **B** Diagram of *GFP*-KI donor construct with multiple sgRNAs

To investigate the effects of sgRNAs with different activities on GT events, sequential transformation constructs were generated for *GFP*-KI to two genetic loci using these four single sgRNAs (Fig. 2A). In this study, we used the same donor sequence as previously reported, with the 1 Kbp homology arms (Additional file 1: FigS1) [32]. The generated *EMB2410*-*GFP* and *ROS1*-*GFP* GT constructs were transformed into the DD45 promoter-driven Cas9-expressing parental line (ABRC stock CS69955) by *Agrobacterium tumefaciens* (Agrobacterium) flower dipping method.

To analyze GT events, five different primer sets were designed for PCR based genotyping (Fig. 2A). The 5′ and 3′ arms specific primers were designed to specifically detect *GFP*-KI events. The 5′ and 3′ external primer sets were designed to contain a primer external of the homology arm and another one within the another homology arm sequence, theoretically capable to amplify both the endogenous and KI alleles. The last one, full-length primers were designed to anneal to the upstream and downstream of the homology arms, capable of amplifying endogenous and precise KI alleles. The specific and external primer sets were used for initial screening, and then all possible T1 candidate plants were characterized by using full-length primer set to assess precise GT. The all GT events obtained with the full-length primer set are precise and stably inherited by offspring [12, 32, 38].

**Fig. 3:**
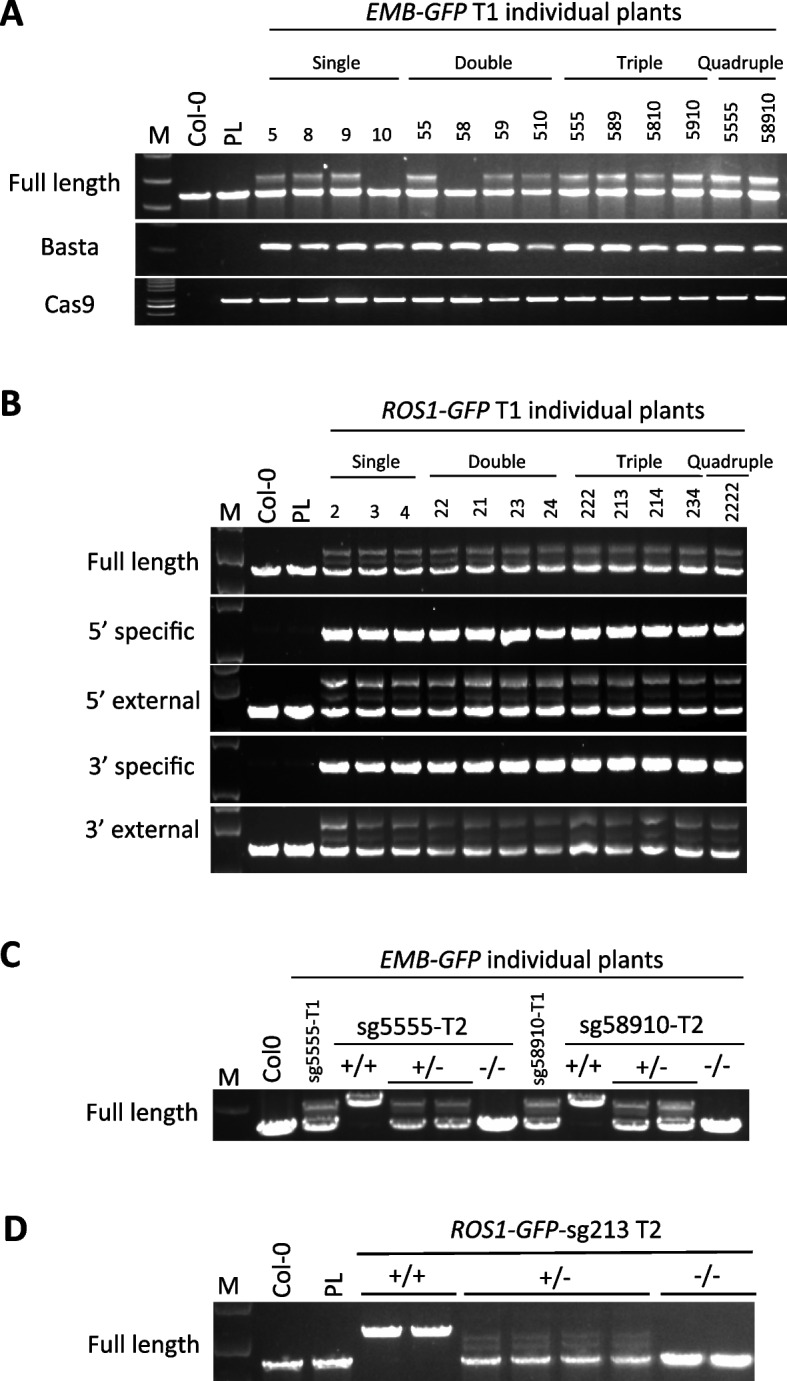
Genotyping of *GFP*-KI GT events with sgRNA combinations. **A**, **B** PCR genotyping of precise *GFP*-KI for the *EMB2410* (**A**) and *ROS1* (**B**) loci in T1 transformants. **C**, **D** PCR genotyping of precise GT of the *EMB2410-GFP-*sg5555 and *EMB2410-GFP-*sg58910 (**C**) and *ROS1-GFP-*sg213 (**D**) in T2 generation. M; marker, PL; parental line. Single means the single sgRNA donor construct; Double, Triple, Quadruple mean *GFP*-KI donor construct with multiple sgRNAs respectively

PCR analysis of a total of 139 to 357 T1 transformants detected at least one precise GT-positive plant using *EMB*-sgRNA5, *EMB*-sgRNA8, and *EMB*-sgRNA9, but not *EMB*-sgRNA10 (Fig. 3A). The GT efficiency of precise *GFP*-KI was highest for *EMB-GFP*-sg9 at 1.5%; the others were 0.5% for *EMB-GFP*-sg5 and 0.3% for *EMB-GFP*-sg8 (Table 1). These in-frame *GFP*-KI events at the *EMB* locus were confirmed by Sanger sequencing (Additional file 1: FigS1). Similarly, at least one precise *GFP*-KI was detected at the *ROS1* locus from 188 to 300 T1 transgenic plants, although it was not detected in *ROS1*-*GFP-s*g1 (Fig. 3B). *ROS1-GFP*-sg2 had the highest precise GT efficiency of 3.2%, followed by *ROS1-GFP*-sg3 with 1.6% and *ROS1-GFP*-sg4 with 0.5% (Table 1).

**Table 1:** Knock-in GT efficiencies at the *EMB2410* and *ROS1* loci

	Construct		Number of transformants analyzed	Total GT	Total GT efficiency (%)	Precise GT (biallelic)	Precise GT efficiency (%)
*EMB-GFP*	Single sgRNA	*EMB-GFP-*sg5	192	9	4.7	1	0.5
		*EMB-GFP*-sg8	357	15	4.2	1	0.3
		*EMB-GFP*-sg9	198	9	4.5	3	1.5
		*EMB-GFP*-sg10	139	0	0	0	0
	Double sgRNAs	*EMB-GFP*-sg55	380	18	4.7	1	0.3
		*EMB-GFP*-sg58	197	61	31.0	0	0
		*EMB-GFP*-sg59	217	51	23.5	4	1.8
		*EMB-GFP*-sg510	171	20	11.7	3	1.8
	Triple sgRNAs	*EMB-GFP*-sg555	226	40	17.7	5	2.2
		*EMB-GFP*-sg589	194	62	32.0	2	1.0
		*EMB-GFP*-sg5810	233	20	8.6	1	0.4
		*EMB-GFP*-sg5910	228	13	5.7	1	0.4
	Quadruple sgRNAs	*EMB-GFP*-sg5555	202	34	16.8	3 (1)	1.5
		*EMB-GFP*-sg58910	167	52	31.1	4	2.4
*ROS1-GFP*	Single sgRNA	*ROS1-GFP-*sg1	300	0	0	0	0
		*ROS1-GFP-*sg2	222	35	15.8	7	3.2
		*ROS1-GFP-*sg3	188	21	11.2	3	1.6
		*ROS1-GFP-*sg4	218	9	4.1	1	0.5
	Double sgRNAs	*ROS1-GFP-*sg22	408	30	7.4	5	1.2
		*ROS1-GFP-*sg21	204	41	20.1	4	2.0
		*ROS1-GFP-*sg23	461	57	12.3	5	1.1
		*ROS1-GFP-*sg24	408	51	12.5	4	1.0
	Triple sgRNAs	*ROS1-GFP-*sg222	286	50	17.5	2	0.7
		*ROS1-GFP-*sg213	394	55	14.0	1	0.3
		*ROS1-GFP-*sg214	226	21	9.7	2	0.9
		*ROS1-GFP-*sg234	276	31	11.2	1	0.4
	Quadruple sgRNAs	*ROS1-GFP-*sg2222	263	37	14.1	1	0.4
		*ROS1-GFP-*sg2341	429	40	9.3	0	0

GT efficiency was calculated based on the number of individual T1 transformants examined

**Fig. 4:**
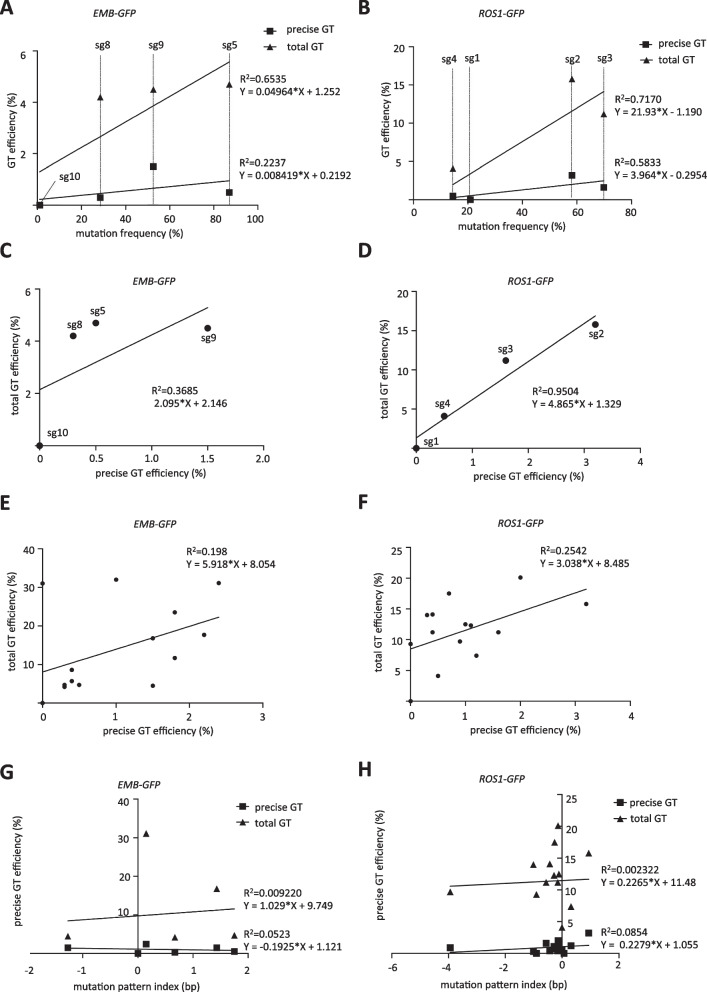
Correlation analysis of mutation frequency and GT efficiency. **A**, **B** Correlation analysis of mutation frequency and *GFP*-KI GT efficiency by single sgRNA at the *EMB2410* (**A**) and *ROS1* (**B**) loci. Squares indicate precise GT, triangles indicate total GT events. **C**, **D** Correlation analysis of precise and total GT efficiency by single sgRNA for the *EMB2410* (**C**) and *ROS1* (**D**) loci. The four filled circles represent four different single sgRNA donor constructs. **E**, **F** Correlation analysis for all precise and total GT events at the *EMB2410* (**E**) and *ROS1* (**F**) loci. A total of fourteen *GFP*-KI donor constructs with single or multiple sgRNAs were analyzed for each of the two loci. **G**, **H** Correlation analysis of mutation pattern and *GFP*-KI GT efficiency at the *EMB2410* (**G**) and *ROS1* (**H**) loci. Squares indicate precise GT; triangles indicate total GT events

Statistical analysis of the correlation between mutation frequency and precise GT efficiency revealed a linear relationship of *R*^2^ = 0.2237 and *R*^2^ = 0.5833 for the *EMB2410* and *ROS1* loci *GFP*-KI, respectively (Fig. 4A, B). During the screening process, not only the precise GT but also the precise GT events with only 5′ arms were determined (Table 1). The precise GT with only 5′ arm region was due to HDR-mediated KI at the 5′ arm region, while the other 3′ arm side was incorporated by NHEJ-mediated repair [32, 39]. Therefore, since at least one side arm was incorporated by the HDR, the 5′ arm precise KI events were considered as total GT events in this study. Correlation analysis between mutation frequency and total GT efficiency showed a much stronger linear relationship with *R*^2^ = 0.6535 and *R*^2^ = 0.7170 for *EMB-GFP* and *ROS1-GFP*, respectively (Fig. 4A, B). Furthermore, analysis of the correlation between precise GT efficiency and total GT efficiency showed a strong linear relationship for *ROS1-GFP* (*R*^2^ = 0.9504), but less so for *EMB-GFP* (*R*^2^ = 0.3685) (Fig. 4C, D). These results suggest that DSBs activity of different sgRNAs contributes to GT efficiency and is one of the important factors for GT efficiency. As the mutation frequency increases, the total efficiency of GT tends to increase. Nevertheless, this does not imply an exact increase in GT efficiency. This indicates that increasing the DSBs activity at the target site can improve the overall probability of HDR occurrence.

### Enhancing GT efficiency through multiple-sgRNA strategy

On the basis of above results, we hypothesized that the simultaneous application of multiple sgRNAs would increase the occurrence of DSBs at the same target site, which would in turn increase the likelihood of HDR-mediated GT events. Indeed, highly efficient mutagenesis has been observed by applying multiple different sgRNAs to the target site [34, 36], but to our knowledge, increasing the copy number of the same sgRNA has not been attempted in plants. However, it has been reported that strong expression of sgRNAs is crucial for efficient mutagenesis in plants [35]. Expression cassettes containing the same or different combinations of sgRNAs and *GFP*-KI donor constructs were generated to explore the effects of multiple sgRNAs on CRISPR/Cas9-mediated GT efficiency (Fig. 2B). A total of 10 donor constructs were generated for each target gene by combining 2 to 4 sgRNAs (Additional file 1: FigS2). The constructs were based on sgRNA5 for *EMB2410* and sgRNA2 for *ROS1* because these two sgRNAs were used in our previous research and were retained to provide a basic guarantee of GT efficiency [32] (Fig. 2B, Additional file 1: FigS2).

Studies to increase the number and types of sgRNA expression cassettes yielded at least one precise *GFP*-KI GT event in all combinations, except the double and quadruple different sgRNAs combination constructs *EMB-GFP*-sg58 and *ROS1-GFP*-sg2341 (Fig. 3A, B, Table 1). All GT events detected by the full-length primer set were inherited by offspring at Mendelian ratios (Fig. 3C, D). This result is consistent with previous reports [12, 32, 38]. Increasing the number of the same sgRNA enhanced GT efficiency in *EMB2410* but did not affect the GT efficiency in *ROS1*, which actually decreased GT efficiency. On the other hand, in all cases except the double sgRNAs *ROS1-GFP*-sg22, the total GT efficiency was substantially increased compared to single sgRNA. These results indicate that increasing the number of sgRNAs can promote GT efficiency in plants.

Combinations of double, triple, and quadruple different sgRNAs do not seem to significantly improve the precise *GFP*-KI efficiency as expected but can maintain relatively high efficiency throughout the total GT events (Table 1). Total GT efficiency was 3- to 6.8-fold higher with the double sgRNAs combinations of *EMB-GFP*-sg58, *EMB-GFP*-sg59, and *EMB-GFP*-sg510 compared to the single sgRNA *EMB-GFP*-sg5. Furthermore, the GT efficiency of the precise KI of *EMB-GFP*-sg59 and *EMB-GFP*-sg510 was superior to that of single sgRNA constructs. The triple sgRNAs combination *EMB-GFP*-sg589 increased total GT efficiency by 7.1-fold and full-length precision KI efficiency by twofold compared to the single sgRNA *EMB-GFP*-sg5. The additional usage of *EMB-*sgRNA10 did not improve performance beyond using the efficient single sgRNA but was still better than *EMB-GFP*-sg10. The use of the quadruple sgRNAs construct *EMB-GFP*-sg58910 resulted in the highest precise KI efficiency of all *EMB-GFP* constructs identified. The application of multiple sgRNAs at the *EMB2410* locus improved the overall HDR event frequency and thus the GT efficiency.

GT events mediated by the double sgRNAs donor constructs, *ROS1-GFP*-sg21 and *ROS1-GFP*-sg24, achieved total GT efficiencies of 20.1% and 12.5% and precise GT efficiencies of 2.0% and 1.0%, respectively (Table 1). This demonstrates that the GT efficiency obtained by the double sgRNAs is improved compared to the GT efficiency by *ROS1-GFP*-sg1 and *ROS1-GFP*-sg4, indicating that the combination of sgRNAs strategy can improve the efficiency of ineffective sgRNA-mediated GT events. On the other hand, the double sgRNAs construct *ROS1-GFP*-sg23 showed no significant improvement in GT efficiency compared to the single sgRNA constructs. The total GT efficiencies of the triple sgRNAs constructs *ROS1-GFP*-sg213, *ROS1-GFP*-sg214, and *ROS1-GFP*-sg234 were 14.0%, 9.7%, and 11.2%, respectively, with precise GT efficiencies of 0.3%, 0.9%, and 0.4%, respectively. Interestingly, the application of multiple-sgRNA strategy at the *ROS1* locus resulted in a lower efficiency of the precise GT events compared to the utilization of single sgRNA strategy.

Statistical analysis was performed for all GT events. Analysis of the correlation between all precise GT efficiency and all total GT efficiency revealed a weak positive linear relationship for *EMB-GFP* (*R*^2^ = 0.198) and *ROS1-GFP* (*R*^2^ = 0.2542) (Fig. 4E, F). These results suggest that the efficiency of total GT events represents the trend in precise GT efficiency.

### Multiple-sgRNA strategy elevates both of mutation frequency and sgRNAs expression

**Fig. 5:**
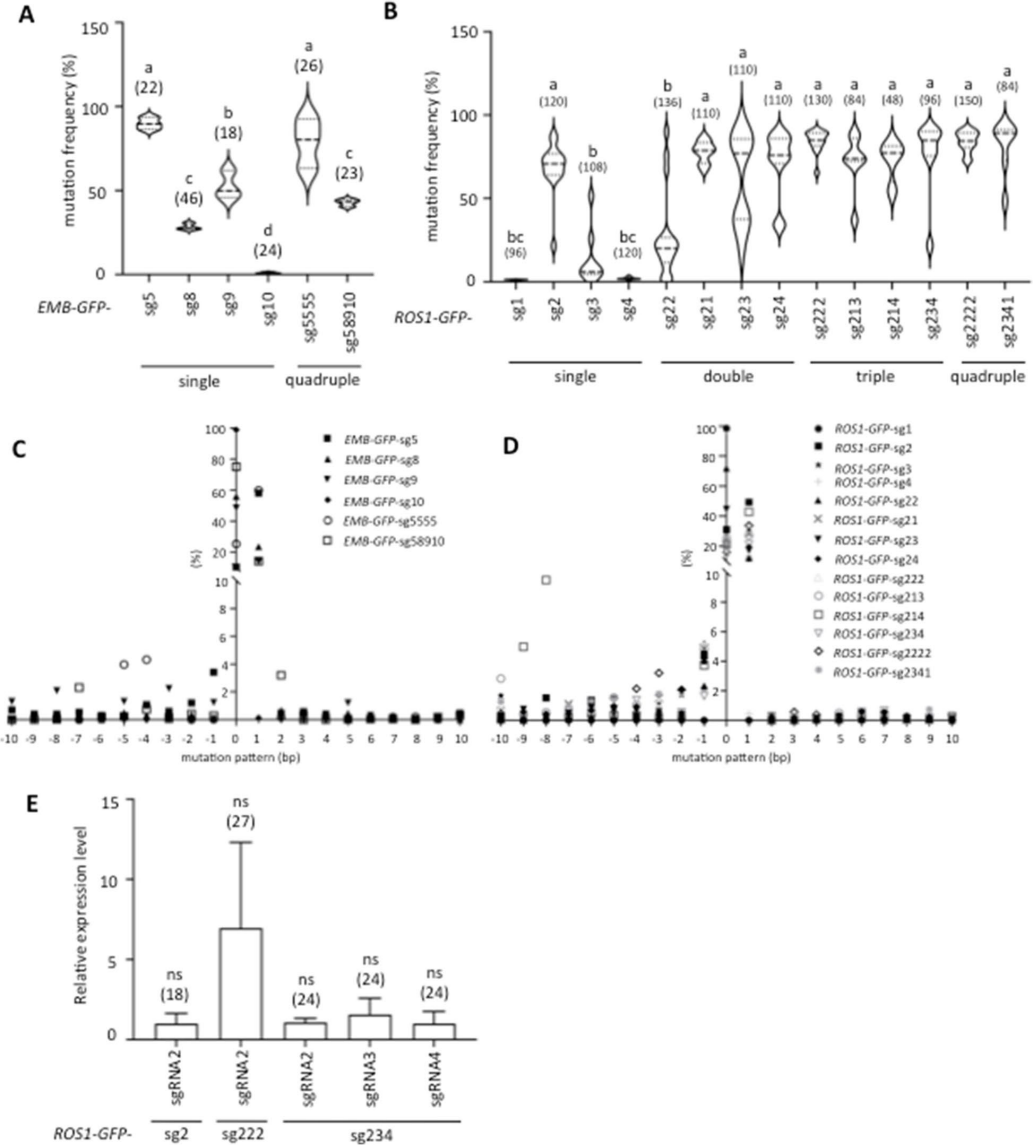
DSB activity and expression of sgRNAs. **A** Mutation frequency in GT-negative *EMB-GFP* KI transgenic plants. Mutation frequencies at *EMB2410* target sites were analyzed in different GT-negative *EMB-GFP* T1 transgenic plants. **B** Mutation frequency in GT-negative *ROS1-GFP* KI transgenic plants. Mutation frequencies at *ROS1* target sites were analyzed in the GT-negative *ROS1-GFP* T1 transgenic plants. **C**, **D** Mutation patterns in GT-negative *EMB-GFP* (**C**) and *ROS1-GFP* (**D**) KI transgenic plants. The relationship between average mutation pattern (bp) (Additional file 3: Table S3) and GT efficiency is shown. **E** Expression analysis of *ROS1* sgRNA. Expression levels of sgRNAs targeting *ROS1* were determined by qRT-PCR in *ROS1-GFP* T1 transgenic plants. *ROS1*-sgRNA2 expression was determined in single-sgRNA *ROS1-GFP*-sg2, triple-sgRNA *ROS1-GFP*-sg222, *ROS1-GFP*-sg234. *ROS1*-sgRNA3 and *ROS1*-sgRNA4 were analyzed in different triple sgRNAs *ROS1-GFP*-sg234 T1 transgenic plants. *Actin7* gene (At5g09810) was used as an internal reference gene. The numbers in parenthesis represent the number of individual samples analyzed. ns; not significant. One way ANOVA and Tukey test were used for difference analysis (*P* < 0.05)

To facilitate the effective use of multiple-sgRNA strategy, it is crucial to define the combination of different sgRNA-specific editing features. In order to investigate why the use of multiple sgRNAs in combination did not drastically improve the efficiency of overall and precise GT events, we measured CRISPR/Cas9-mediated mutation frequencies at *EMB2410* and *ROS1* target loci. A total of 18 to 150 *GFP*-KI negative T1 transformants for each construct were analyzed to determine their mutation frequencies and patterns. Mutation frequencies by single sgRNA constructs (*EMB-GFP*-sg5/8/9/10) for *GFP*-KI were similar to the analysis of Col-0 background (*EMB*-sgRNA5/8/9/10) (Fig. 1D, Fig. 5A). Increasing the copy number of *EMB*-sgRNA5 (*EMB-GFP*-sg5555) did not change the mutation frequency from that of single sgRNA *EMB-GFP*-sg5 construct. Mutation frequency in the combination of different quadruple sgRNAs *EMB-GFP*-sg58910 was higher than in *EMB-GFP*-sg8 and *EMB-GFP*-sg10 but slightly lower than in *EMB-GFP*-sg5 and *EMB-GFP*-sg9.

Sequencing results showed that the mutation frequencies of *ROS1-GFP*-sg1, *ROS1-GFP*-sg2, and *ROS1-GFP*-sg4 containing single sgRNA were almost identical to those with Col-0 as background (Fig. 1E). However, the mutation frequency of *ROS1-GFP*-sg3 was not at all consistent with the result using Col-0 as background (Fig. 5B). *ROS1-GFP*-sg22 showed a lower mutation frequency than *ROS1-GFP*-sg2, which is consistent with the result that *ROS1-GFP*-sg22 has a lower GT efficiency than *ROS1-GFP*-sg2. Furthermore, the mutation frequency induced by combinations of double, triple, and quadruple sgRNAs was significantly higher than that induced by single *ROS1-*sgRNA1, *ROS1-*sgRNA3, and *ROS1-*sgRNA4, but statistical analysis showed no significant difference in the mutation frequency induced by multiple copies of *ROS1*-sgRNA2 constructs (Fig. 5B).

In addition to mutation frequency, mutation patterns were also analyzed. The predominant mutation pattern is a single base insertion followed by a single base deletion in all sgRNAs and combinations of them (Fig. 5C, D, Additional file 1: FigS3). When multiple different sgRNAs were applied, the frequency of long deletions was elevated. To clarify the relationship between mutation patterns and GT efficiency, we examined averaged mutation patterns and GT efficiency. The results indicate that at least the average mutation pattern is not associated with the GT efficiency of *GFP*-KI at the *EMB2410* and *ROS1* loci (Fig. 4G, H). However, this is an indirect experiment since the mutation patterns were determined in GT-negative plant samples. Thus, there is no direct evidence as to what mutation patterns are optimal for efficient GT, and further research is warranted.

Next, the relative sgRNA expression level in 18 to 27 independent T1 transgenic plants was also measured and analyzed. The average expression level of *ROS1*-sgRNA2 was increased approximately sixfold in *ROS1-GFP*-sg222 compared to *ROS1-GFP*-sg2, but this was not statistically significant (Fig. 5E). The variance of independent T1 proportions is likely responsible for the lack of statistically differences. In contrast, *ROS1*-sgRNA2 expression was comparable between single-sgRNA *ROS1*-GFP-sg2 and triple-sgRNA *ROS1-GFP*-sg234 transgenic plants (Fig. 5E). Furthermore, expression levels of *ROS1-*sgRNA2, *ROS1*-sgRNA3, and *ROS1*-sgRNA4 were nearly identical in T1 generation plants of the *ROS1-GFP-*sg234 triple sgRNAs with the donor construct (Fig. 5E). These results suggest that sgRNA expression levels correlate with expression cassette dose and that tandem repeats of the AtU6-26 promoter expression cassette do not cause transcriptional silencing. These results are consistent with previous report [40].

### Dosage of sgRNAs do not influence off-targeting

**Fig. 6:**
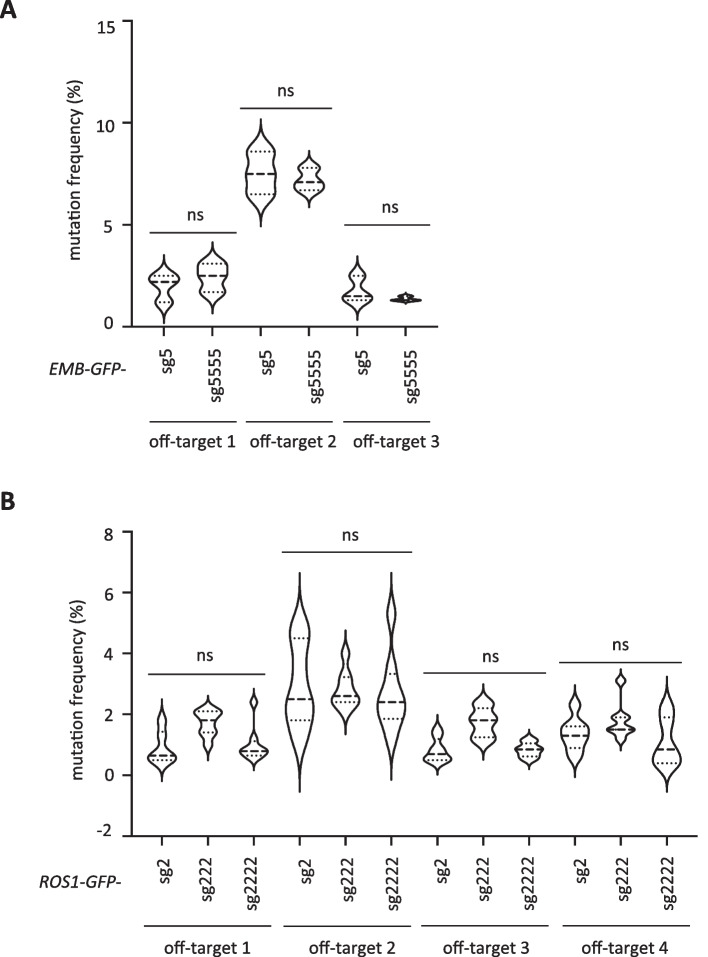
Off-target effects of multiple sgRNAs in Arabidopsis. **A**, **B** Mutation frequency of possible off-target sites in *EMB-GFP* (**A**) and *ROS1-GFP* (**B**) KI transgenic plants. Mutation frequencies of possible off-target sites (Additional file 3: Table S4, S5) were analyzed by PCR followed by sequencing. ns, not significant. One way ANOVA and Tukey test were used for difference analysis between data (*P* < 0.05)

The main cause of off-targeting by the CRISPR/Cas system is thought to be the massive amount of Cas protein and sgRNA in the cell [41]. As mentioned above, excessive amounts of sgRNA expression were detected in this study (Fig. 5E). Therefore, when applying the strategy of using multiple sgRNAs, there was concern that increasing the copy number of sgRNA would have an off-target effect on the plant. To address this question, we employed a website database tool that predicts possible off-target sites for each sgRNA, selected the top 3–4 potential off-target sites, and examined them by Sanger sequencing (Additional file 3: Table S4). The results showed that the multiple-sgRNA strategy did not differ in mutation frequency at all off-target sites compared to the use of single sgRNA (Fig. 6, Additional file 3: Table S5). It is suggested that by increasing the copy number of sgRNA or incorporating different sgRNAs, genome editing outcomes comparable to those achieved with single sgRNA can be obtained without unwanted targeted mutations.

### Imprecise GT events

This study detected not only precise GT events but also imprecise heritable GT events for all GT constructs (Table 1). Most of these imprecise GTs were detected as bands of larger size than expected (Additional file 1: FigS3A). This indicates that the imprecise GT events are due to the insertion of unwanted sequences. To investigate imprecise GT events, Sanger sequencing was performed on the PCR products of the specific primer set for the 5′ arm of *ROS1*-*GFP*. As a result, partial T-DNA insertion from the donor *GFP* into the RB sequence was detected in the *ROS1*-*GFP* line with an imprecise 5′ homology arm integration (Additional file 1: FigS3B). This indicates that one homology arm was precisely incorporated via HDR and the site of the other homology arm was inserted by NHEJ with T-DNA. These observations are consistent with previous study [32]. These results strongly suggest that the single-stranded T-DNA released from the binary vector by Agrobacterium is likely to be the repair donor template for GT in this system.

## Discussion

In this study, we verified a less strong but linear relationship between DSBs activity of Cas9 and GT efficiency at the *EMB2410* and *ROS1* loci when single sgRNA was applied. Previously, we examined 27 different endogenous loci in Arabidopsis and concluded that DSBs frequency via the CRISPR/Cas9 system is associated with GT efficiency [32]. Alternatively, this study revealed a correlation between DSBs and GT by using four different sgRNAs targeting the same region with the same donor template at two different loci. Our results clearly demonstrate the importance of sgRNA design for efficient GT and are consistent with previous studies [32].

It is widely recognized that sgRNA design has a significant impact on genome editing efficiency [42]. Because there may not be a strong correlation between actual efficiency and predicted score, relying solely on the predicted sgRNA score of the target gene sequence is not always reliable. One of the key issues in achieving highly efficient CRISPR/Cas9-mediated GT is how to select efficient sgRNAs and how to avoid inefficient impacts of sgRNAs. The importance of sgRNA expression levels and combinations of different sgRNAs for efficient mutagenesis has already been reported in plants [34, 35]. In principle, multiple sgRNAs can be applied to efficiently generate DSBs and short deletions to initiate HDR [33, 36, 37]. In addition, combinations of different sgRNAs targeting the same region can compensate each other, even if the selected sgRNA pool contains inefficient sgRNAs. Moreover, even after an allele has been mutated by NHEJ, its target site can still be targeted by other sgRNAs, similar to secondary sgRNAs in the “double tap” method [43]. Therefore, the use of multiple sgRNAs can theoretically increase the frequency of HDR occurrence.

Our results showed that the GT efficiency of *GFP*-KI was improved, especially when multiple sgRNAs were applied to the *EMB2410* locus. The main reason for the increase in GT efficiency could be attributed to the higher DSBs frequency due to multiple sgRNAs. GT efficiency was improved for *EMB2410* when triple and quadruple sgRNAs were used, but not when double sgRNAs were applied. It is hypothesized that the amount of sgRNA expression may cause a change in editing efficiency only after a certain threshold is reached [22, 44]. Another possibility may be due to the ability of some sgRNAs to alter the chromosomal microenvironment or to compete effectively with active sgRNAs [29, 45–47]. The double sgRNAs construct of *EMB-GFP*-sg510 achieved a higher GT, while other combinations including *EMB-GFP*-sg10 showed lower editing efficiency. In the case of *EMB-GFP*-sg510, *EMB*-sgRNA10 may have a similar role as a proxy sgRNA. Proxy sgRNA consists of 14- or 15-nt protospacer sequences and can lead Cas9 to the target site without DSB activity which can loosen chromatin structure and promote Cas9-mediated DSBs [48, 49]. Such higher DSBs and/or chromatin opening by proxy sgRNAs may also enhance the subsequent HDR activity. In some cases, combinations of sgRNAs may be more advantageous in genome editing than inefficient sgRNAs alone.

In contrast, a portion donor constructs of *EMB2410* and the majority of *ROS1* did not show dramatic improvement in GT efficiency with the multiple-sgRNA strategy. The sgRNAs designed in this study are almost overlapped and thus may compete with each other. The application of multiple sgRNAs may result in the accumulation of more sgRNA-Cas9 complexes at the target site, inhibiting the binding of factors involved in the HDR pathway and delivery of the donor template to the DSBs site [44]. The most likely explanation is that the DSBs needed for efficient GT may already be saturated. It has been reported that the relationship between DSBs activity and HDR occurrence is not always linear; excessive frequencies of DSBs may be redundant for HDR [44].

In the present study, the size of the deletion does not appear to play an important role in HDR efficiency rather than the DSB frequency itself. In the mouse cell cultures and zygotes experimental system, short deletions by overlapping sgRNAs design promoted GT efficiency. In contrast, when long deletions were generated by non-overlapping sgRNAs, HDR-mediated donor sequence integration efficiency was dramatically decreased [36]. Thus, it suggests that the HDR mechanism may be more predominant when short deletions due to overlapping sgRNAs occur [36, 37]. However, previous reports have employed single-stranded oligo donor templates with short (45–60 nt) homologous arms [36]. We speculate from this report that the size of the deletion may affect the recognition efficiency of the homologous arm and subsequent GT. Since we used donor constructs with 1 Kbp long homology arms, the size of the deletion might not affect GT efficiency in this study.

Another important question is whether the distance between the target DSB site of the sgRNA and the location of the *GFP*-KI affects GT efficiency. It is reported that the intended base substitutions in the homologous arms near the DSB site were introduced at some target genes but not at other loci [32]. This suggests that the distance between the DSB and GT sites may affect GT efficiency. In this study, all possible DSB sites are located between the 5′ and 3′ homology arms, except for *ROS1*-sgRNA3. And our results show that DSB efficiency is the main contributor to GT efficiency. Therefore, more detailed analysis is needed to elucidate the relationship between DSB and GT sites.

The results of this study indicate that there is a linear relationship between the number of sgRNA expression cassettes and the amount of sgRNA expression and that tandem repeats of the same AtU6-26 promoter cassette are not responsible for their transcriptional gene silencing [36]. Although it has not been analyzed in this study, instead of increasing the sgRNA expression cassette, a strong promoter that drives the sgRNA could improve DSB and subsequent GT efficiency [35]. Furthermore, it is revealed that off-targeting effects is very unlikely in plants, even when sgRNA expression levels are increased [50–52]. However, the expression of Cas9 does not seem to be very high in the parental line due to the silencing of the transgene [32]. In other words, even if the expression level of the sgRNA is increased further, the functional sgRNA-Cas9 complex will reach a saturation state. Therefore, the expression level of Cas9 must be increased at the same time to increase the functional complex. Further analysis of the relationship between the amount of functional Cas9 complexes and off-target in plants is needed.

We have previously shown that T-DNA copy number and transgene expression are not associated with GT efficiency [12, 31, 32, 38]. On the other hand, this study revealed that GT efficiency can be improved by increasing the number of sgRNA cassettes. In previous reports, T-DNA copy number and transgene expression were analyzed among individual transgenic lines of a construct. Therefore, the effects of actual sgRNA copy number and expression may be covered by other factors, such as the positional effects of T-DNA integration. In contrast, the present study examined the effects of different sgRNA copy numbers across constructs, which would have allowed us to detect these effects unambiguously.

## Conclusions

This study provides novel insights into the relationship between sgRNA, DSB, and GT. The use of multiple-sgRNA strategy contributes to improved efficiency of GT while compensating for inefficient results caused by inefficient sgRNAs. Utilize multiple sgRNAs are able to improve DSBs and total GT efficiencies, but this not always linked with precise GT efficiency. This finding strongly suggests that some additional innovations, such as HDR machinery, as well as DSB frequency, are needed to improve precise GT efficiency. According to the results of this study, including DSB and GT efficiency, three sgRNA cassettes appear to be optimal for efficient GT. Further research is necessary to optimize the design of multiple sgRNAs and determine how to maximize its efficacy of GT. Our findings will be useful in advancing the field of genome editing in plants.

## Methods

### Plant materials and growth condition

The *Arabidopsis thaliana* (Arabidopsis) accession Col-0 and Arabidopsis Biological Resource Center (ABRC) donated the stock number CS69955 parental line, previously named DD45-#58 [12, 32], were used for all experiments. The seeds were sterilized with sodium hypochlorite. The plants of Col-0 and the parental line were grown at 22 °C on half Murashige and Skoog (MS) medium for 10 to 14 days and transplanted to soil under a photoperiod of a 16-h light and 8-h dark. A soil mixture of peatmoss, vermiculite, and perlite in a 2:1:1 ratio with a pH of approximately 6.5 was used to grow the plants. A balanced liquid fertilizer was applied every 2 weeks.

### Plasmid construction

For DSB frequency and GT via sequential transformation strategy, the destination vectors were constructed according to published protocols [12, 53]. Briefly, sgRNAs were designed by using online websites CRISPOR (http://crispor.tefor.net/), CRISPR Primer Designer (http://plantsignal.cn/CRISPR/crispr_primer_designer.html), CRISPR-PLANT (https://www.genome.arizona.edu/crispr/index.html), CHOPCHOP (http://chopchop.cbu.uib.no/), DESKGEN (https://www.deskgen.com/landing/cloud.html), and CRISPR tool ATUM (https://www.atum.bio/eCommerce/cas9/input); AtU6-26 promoter driven sgRNA cassette and donor sequence were constructed in pCambia3301. The *GFP*-KI donors were used with the 1 Kbp homology arms, as previously reported (Additional file 1: FigS1) [12, 32]. Ligation products were transformed into *E. coli*, monoclonal bacteria were detected by PCR, recombinant plasmids were extracted and sequenced.

### Arabidopsis plant transformation

The generated constructs were transformed into *Agrobacterium tumefaciens* (Agrobacterium) GV3101 strain competent cells by heat shock method and cultured on LB solid medium containing kanamycin and rifampicin, incubated in the dark at 28 °C for 2 days to obtain positive colonies. The transformed Agrobacterium is pre-cultured in the 5 mL liquid LB, then grew in a large culture with 150 ml LB, and then collected by centrifugation at 4000 rpm for 20 min. The collected Agrobacterium was resuspended in an infection solution containing 5% (w/v) sucrose, 0.22% (w/v) MS, and 0.05% (v/v) Silwet-77. Cut off all fruit pods and white flowers from the plants the night before or on the day of transformation. Soak the plant buds in the infection solution for 45 s, remove and shake gently, then wrap in plastic wrap to maintain humidity. The plants were placed in darkness at 22 °C for 20 h. The wrapping was removed, and the plants were transferred to normal growth conditions.

For screening with hygromycin, T1 seeds were grown on 1/2 MS plates containing 50 mg/L hygromycin. Surviving seedlings were transplanted into soil and further cultured for genotyping. The new transformants of sequential transformation T1 lines were directly sowed in soil and then screened by spraying three times with Basta at a concentration of 0.2% (v/v).

### DNA analysis

The total genomic DNA was extracted from leaf tissue by cethyltrimethyl ammonium bromide (CTAB) method for individual plant analysis. Leaf tissues were ground to fine powder in liquid nitrogen using the ShakeMaster AUTO (Bio Medical Science Inc., Tokyo, Japan). The extracted DNA was used for PCR analysis of GT events. Specific primers were designed for genotyping (Additional file 3: Table S4). The PCR system used 2 × Taq Plus Master Mix II (Vazyme, Nanjing, China), according to the instructions. The PCR products were separated by electrophoresis on 1.5% (w/v) agarose gel and visualized by Image Lab software (Bio-Rad Laboratories, Hercules, USA).

The T7 endonuclease I (T7EI) assay was employed for mutation frequency analysis of T1 individual transgenetic plants. The reaction solution was prepared and denatured at 95 °C for 5 min. The reaction system was then renatured at room temperature, and 0.2 μL of T7EI (New England Biolabs, Massachusetts, USA) was added to the mixture and allowed to react at 37 °C for 30 min. The digestion was analyzed by 2% agarose gel electrophoresis. The value of the mutation frequency was calculated on the basis of the strength of the bands with the aid of Image Lab software.

The determination of the mutation frequency of the target sites was by means of the TIDE (https://tide.nki.nl) website. Ten to 20 T1 transformants of the materials were selected as an analysis sample. The PCR amplicons of the target sites were sequenced. Statistical analysis was performed to determine the mutation frequency by comparing the results with the wild-type sample.

### Quantitative real-time PCR

The total RNA was extracted by Trizol (Invitrogen, Massachusetts, USA) method and dissolved with RNase-free water. Hiscript II Reverse Transcriptase Kit (Vazyme, Nanjing, China) was used for reverse transcription of 1 μg of RNA to obtain cDNA from the samples. Sample cDNA was diluted tenfold as template, and SYBR Green Super Mix (Bio-Rad Laboratories, Hercules, USA) was used for the qPCR reaction system. *Actin7* gene (At5g09810) was used as an internal reference gene to calibrate the expression of target genes. The relative expression of the target gene was calculated by the 2^−∆∆CT^ method.

### Supplementary Information

**Additional file 1: Fig S1.** Sequencing results representing the precise *GFP*-KI of the *EMB2410* and *ROS1* loci in T1 transgenic plants. Diagrams represent detailed *GFP*-KI sites for *EMB2410* (A) and *ROS1* (B) loci. Sequencing chromatograms show the precise *GFP*-KI GT events in the T1 generation as determined by Sanger sequencing. **Fig S2.** Detailed diagram of *GFP* knock-in donor constructs with multiple sgRNAs. ** Fig S3.** Detailed characterization of imprecise GT events. A, Genotyping of imprecise GT events in *ROS1*-*GFP*-sg24. Both precise and imprecise GT events were detected by the 5′ arm specific primers. Heritable and imprecise GT events were observed in the 5′ homologous arm region in three independent *ROS1-GFP-*sg24 plants of the T2 generation. B, Chromatograms of Sanger sequence results. Protospacer-adjacent motif (PAM) are indicated in red letters, mutations are indicated in small green letters.

**Additional file 2: Table S1.** Prediction scores for *EMB2410* and *ROS1* sgRNAs. The sgRNAs activities were predicted by using CRISPOR online website (http://crispor.tefor.net/). **Table S2.** Other candidate sgRNAs for *EMB2410* and *ROS1* loci. The mutation efficiencies were noted by CRISPROR.

**Additional file 3: Table S3.** Mutation pattern. The percentage of mutation patterns were analyzed in different GT-negative *GFP* T1 transgenic plants referring to the *EMB-*sgRNA5 and *ROS1-*sgRAN2 target locus, negative values represent the number of deleted bases and positive values represent the number of inserted bases. **Table S4.** Predicted off-target loci. The off-target loci were predicted by using CRISPR-GE online website (http://skl.scau.edu.cn). **Table S5.** Mutation frequencies of predicted off-target sites. Mutation frequencies were analyzed by PCR followed by sequencing using the T1 transformants. **Table S6.** Sequences of primers.

**Additional file 4: Fig S4.** Original uncropped gel pictures.

## Data Availability

The authors declare that all the data supporting the findings of this study are available within the paper and its Additional files. The data sets generated or analyzed during the current study are available from the corresponding author on reasonable request.

## Abbreviations

SSN: Sequence-specific nucleases
DSB: Double-strand breaks
NHEJ: Non-homologous end-joining
HDR: Homology-directed repair
KI: Knock-in
GT: Gene targeting
CRISPR/Cas9: Clustered regularly interspaced short palindromic repeat (CRISPR)/CRISPR-associated protein 9
SpCas9: *Streptococcus pyogenes* Cas9
sgRNA: Single guide RNA
PAM: Protospacer adjacent motifs
Arabidopsis: *Arabidopsis thaliana*
EMB2410: *Embryo Defective 2410*
ROS1: *Repressor of Silencing 1*
Agrobacterium: *Agrobacterium tumefaciens*
ABRC: Arabidopsis Biological Resource Center
CTAB: Cethyltrimethyl ammonium bromide
MS: Murashige and Skoog
T7EI: T7 endonuclease I
