# Supplementary material for: Application of multiple sgRNAs boosts efficiency of CRISPR/Cas9-mediated gene targeting in Arabidopsis
Source: BMC Biol. 2024 Jan 17;22:6. doi: 10.1186/s12915-024-01810-7 (PMC10795408; doi:10.1186/s12915-024-01810-7)
Supplement: Supplementary file 1 — Additional file 1: Fig S1. Sequencing results representing the precise GFP-KI of the EMB2410 and ROS1 loci in T1 transgenic plants. Diagrams represent detailed GFP-KI sites for EMB2410 (A) and ROS1 (B) loci. Sequencing chromatograms show the precise GFP-KI GT events in the T1 generation as determined by Sanger sequencing. Fig S2. Detailed diagram of GFP knock-in donor constructs with multiple sgRNAs. Fig S3. Detailed characterization of imprecise GT events. A, Genotyping of imprecise GT events in ROS1-GFP-sg24. Both precise and imprecise GT events were detected by the 5′ arm specific primers. Heritable and imprecise GT events were observed in the 5′ homologous arm region in three independent ROS1-GFP-sg24 plants of the T2 generation. B, Chromatograms of Sanger sequence results. Protospacer-adjacent motif (PAM) are indicated in red letters, mutations are indicated in small green letters. [file 12915_2024_1810_MOESM1_ESM.pdf]

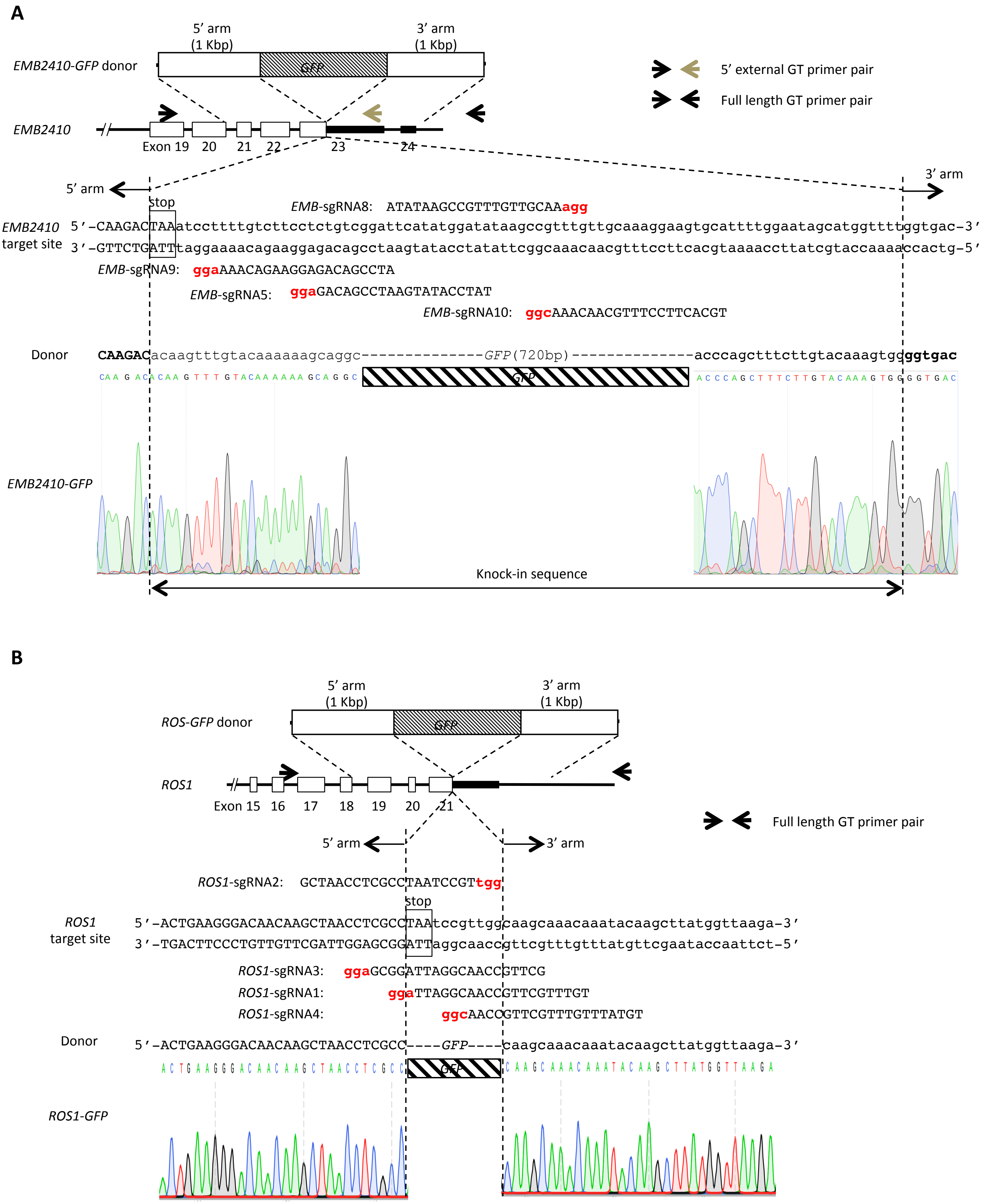

FigS1. Sequencing results representing the precise *GFP*-KI of the *EMB2410* and *ROS1* loci in T1 transgenic plants.

Double sgRNAs

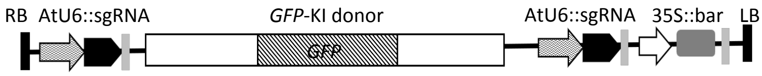

EMB-GFP-sg55	sgRNA5	sgRNA5
EMB-GFP-sg58	sgRNA5	sgRNA8
EMB-GFP-sg59	sgRNA5	sgRNA9
EMB-GFP-sg510	sgRNA5	sgRNA10
ROS1-GFP-sg22	sgRNA2	sgRNA2
ROS1-GFP-sg21	sgRNA2	sgRNA1
ROS1-GFP-sg23	sgRNA2	sgRNA3
ROS1-GFP-sg24	sgRNA2	sgRNA4

Triple sgRNAs

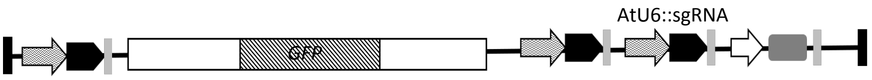

EMB-GFP-sg555	sgRNA5	sgRNA5	sgRNA5
EMB-GFP-sg589	sgRNA5	sgRNA8	sgRNA9
EMB-GFP-sg5810	sgRNA5	sgRNA8	sgRNA10
EMB-GFP-sg5910	sgRNA5	sgRNA9	sgRNA10
ROS1-GFP-sg222	sgRNA2	sgRNA2	sgRNA2
ROS1-GFP-sg213	sgRNA2	sgRNA1	sgRNA3
ROS1-GFP-sg214	sgRNA2	sgRNA1	sgRNA4
ROS1-GFP-sg234	sgRNA2	sgRNA3	sgRNA4

Quadruple sgRNAs

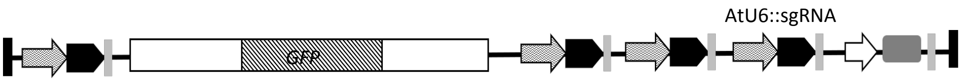

EMB-GFP-sg5555	sgRNA5	sgRNA5	sgRNA5	sgRNA5
EMB-GFP-sg58910	sgRNA5	sgRNA8	sgRNA9	sgRNA10
ROS1-GFP-sg2222	sgRNA2	sgRNA2	sgRNA2	sgRNA2
ROS1-GFP-sg2341	sgRNA2	sgRNA3	sgRNA4	sgRNA1

FigS2. Detailed diagram of *GFP* knock-in donor constructs with multiple sgRNAs.

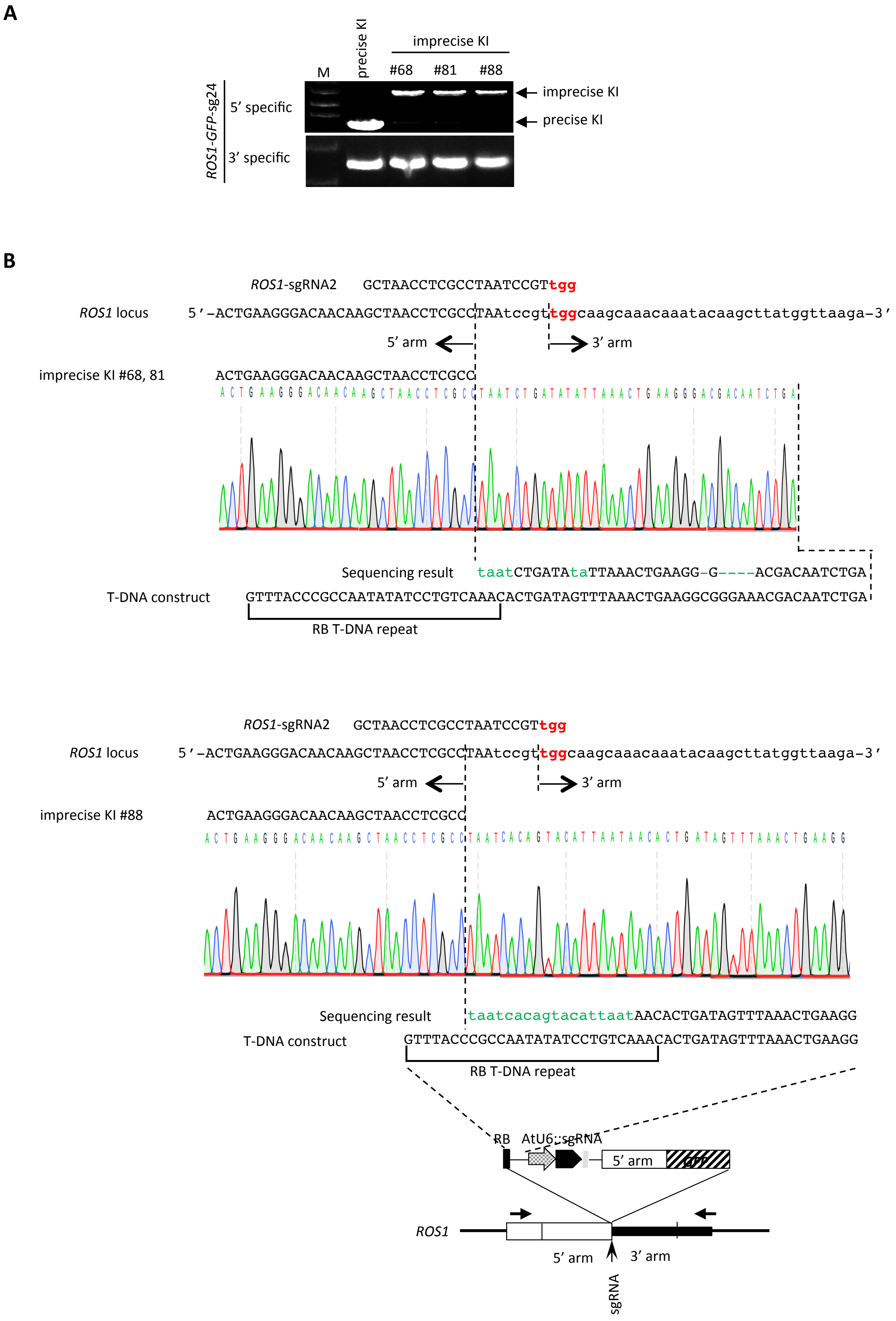

FigS3. Detailed characterization of imprecise GT events.
