# Supplementary material for: Application of multiple sgRNAs boosts efficiency of CRISPR/Cas9-mediated gene targeting in Arabidopsis
Source: BMC Biol. 2024 Jan 17;22:6. doi: 10.1186/s12915-024-01810-7 (PMC10795408; doi:10.1186/s12915-024-01810-7)
Supplement: Supplementary file 2 — Additional file 2: Table S1. Prediction scores for EMB2410 and ROS1 sgRNAs. The sgRNAs activities were predicted by using CRISPOR online website (http://crispor.tefor.net/). Table S2. Other candidate sgRNAs for EMB2410 and ROS1 loci. The mutation efficiencies were noted by CRISPROR. [file 12915_2024_1810_MOESM2_ESM.pdf]

Table S1. Prediction scores for *EMB2410* and *ROS1* sgRNAs.

The sgRNAs activities were predicted by using CRISPOR online website (http://crispor.tefor.net/).

	Mutation frequency (SD)	Precise GT efficiency	Predicted scores by CRISPOR									
			Doench '16	OldDoench '16	Chari	Xu	Wu-Crispr	Doench '14	Wang	Moreno-Mateos	Azimuth in-vitro	CCTop
EMB-sgRNA5	89.9% (±3.6)	0.5%	68	68	86	0.6	72	67	73	65	36	77
EMB-sgRNA8	85.3% (±2.0)	0.3%	58	56	25	-0.2	0	23	59	36	32	56.
EMB-sgRNA9	52.6% (±8.3)	1.5%	51	51	81	0.1	63	19	40	39	35	72
EMB-sgRNA10	1.0% (±0.4)	0%	32	36	14	0.2	56	29	32	47	24	46
ROS1-sgRNA1	20.9% (±11.5)	0%	38	34	19	-0.1	0	2	27	47	32	69
ROS1-sgRNA2	58.2% (±15.5)	3.2%	62	60	64	0.5	0	49	74	52	53	58
ROS1-sgRNA3	69.9% (±13.4)	1.6%	60	65	96	0.8	59	79	86	36	34	78
ROS1-sgRNA4	14.5% (±12.5)	0.5%	59	63	30	0.1	0	52	49	57	34	72

Table S2. Other candidate sgRNAs for *EMB2410* and *ROS1* loci.

The mutation efficiencies were noted by CRISPOR.

	Other candidate sgRNAs	Notes by CRISPOR	Predicted scores by CRISPOR									
			Doench '16	OldDoench '16	Chari	Xu	Wu-Crispr	Doench '14	Wang	Moreno-Mateos	Azimuth in-vitro	CCTop
EMB2410	ATCCTTTTGTCTTCCTCTGTcgg	Not with U6/U3	42	43	0	-0.3	0	8	17	19	43	69
	ATATAAGCCGTTTGTTGCAAagg	/	53	52	5	-0.0	0	20	58	35	44	56
	GTTGCAAAGGAAGTGCATTTtgg	Inefficient	32	29	72	-0.5	0	15	40	34	36	72
	AAGTGCATTTTGGAATAGCAtgg	Not with U6/U3	58	56	25	-0.2	0	23	59	36	32	56
	TTTTGGAATAGCATGGTTTTtgg	Not with U6/U3	40	41	4	-0.5	0	6	39	47	27	75
ROS1	AGCAAACAAATACAAGCTTAtgg	Inefficient	47	40	57	-0.4	0	4	46	27	25	51
