# Supplementary material for: Application of multiple sgRNAs boosts efficiency of CRISPR/Cas9-mediated gene targeting in Arabidopsis
Source: BMC Biol. 2024 Jan 17;22:6. doi: 10.1186/s12915-024-01810-7 (PMC10795408; doi:10.1186/s12915-024-01810-7)
Supplement: Supplementary file 3 — Additional file 3: Table S3. Mutation pattern. The percentage of mutation patterns were analyzed in different GT-negative GFP T1 transgenic plants referring to the EMB-sgRNA5 and ROS1-sgRAN2 target locus, negative values represent the number of deleted bases and positive values represent the number of inserted bases. Table S4. Predicted off-target loci. The off-target loci were predicted by using CRISPR-GE online website (http://skl.scau.edu.cn). Table S5. Mutation frequencies of predicted off-target sites. Mutation frequencies were analyzed by PCR followed by sequencing using the T1 transformants. Table S6. Sequences of primers. [file 12915_2024_1810_MOESM3_ESM.pdf]

Table S3. Mutation pattern.

The percentage of mutation patterns in GT-negative *EMB*-sgRNA5 and *ROS1*-sgRAN2 T1 transgenic plants were analuyzed, negative values representing the number of deleted bases and positive values representing the number of inserted bases.

Mutation pattern percentage of *EMB-GFP* (%)														
Shift range (bp)	sg1	sg2	sg3	sg4									sg5555	sg58910
-10	0.70	0.77	1.30	0.03									0.00	0.00
-9	0.40	0.10	0.37	0.00									0.18	0.00
-8	0.40	0.17	2.07	0.00									0.05	0.03
-7	0.53	0.00	0.27	0.03									0.00	2.30
-6	0.17	0.00	0.30	0.00									0.10	0.20
-5	0.27	0.50	1.30	0.00									3.98	0.23
-4	1.07	0.33	0.93	0.00									4.33	0.73
-3	0.60	0.37	2.23	0.00									0.35	0.08
-2	1.20	0.37	1.20	0.00									0.00	0.40
-1	3.43	0.13	1.23	0.00									0.03	0.28
0	10.43	56.03	48.47	98.97									25.35	75.15
1	58.10	23.60	13.80	0.13									59.85	14.18
2	0.47	0.67	0.17	0.03									0.18	3.20
3	0.57	0.20	0.33	0.00									0.48	0.00
4	0.37	0.33	0.40	0.00									0.28	0.00
5	0.07	0.47	1.20	0.00									0.05	0.03
6	0.03	0.00	0.30	0.00									0.15	0.05
7	0.13	0.00	0.00	0.00									0.18	0.05
8	0.07	0.07	0.00	0.00									0.20	0.00
9	0.00	0.00	0.13	0.03									0.03	0.18
10	0.43	0.00	0.47	0.00									0.40	0.30
Mutation pattern percentage of *ROS1-GFP* (%)														
Shift range (bp)	sg1	sg2	sg3	sg4	sg22	sg21	sg23	sg24	sg222	sg213	sg214	sg234	sg2222	sg2341
-10	0.00	0.15	1.69	0.00	0.11	0.44	0.34	0.30	0.78	2.83	0.08	0.64	0.49	1.26
-9	0.00	0.01	0.62	0.00	0.13	0.45	0.75	0.57	0.72	0.36	4.98	0.40	0.45	0.47
-8	0.00	1.52	0.46	0.00	0.04	0.32	0.09	0.43	0.53	0.55	9.48	0.40	0.42	0.39
-7	0.01	0.49	0.48	0.02	0.09	1.11	0.49	0.42	0.55	0.64	0.20	0.64	0.11	0.99
-6	0.00	1.36	0.64	0.03	0.21	1.08	0.89	0.66	0.72	0.60	0.48	0.81	0.43	1.29
-5	0.00	0.38	0.47	0.02	0.11	0.81	0.67	0.96	0.53	1.55	0.30	1.44	1.47	1.50
-4	0.00	0.26	0.84	0.01	0.23	0.70	0.67	0.95	0.97	1.05	0.32	1.38	2.15	0.77
-3	0.01	0.21	1.09	0.00	0.55	1.50	0.56	0.55	1.26	0.35	0.16	1.05	3.21	1.80
-2	0.00	0.23	1.75	0.01	0.50	0.54	0.37	2.07	2.00	0.40	0.26	0.55	2.07	0.54
-1	0.00	4.38	4.67	0.03	2.35	4.00	4.48	3.98	5.27	4.85	3.72	1.65	5.12	2.14
0	98.69	31.05	21.76	97.82	71.98	28.83	44.52	30.56	15.67	25.40	21.68	23.16	15.81	18.29
1	0.00	49.29	31.03	0.43	12.36	28.31	17.14	19.45	31.85	27.20	42.78	21.20	33.67	11.76

2	0.03	0.33	0.32	0.01	0.36	0.00	0.00	0.04	0.37	0.19	0.00	0.14	0.19	0.37
3	0.00	0.06	0.34	0.01	0.25	0.04	0.02	0.12	0.39	0.18	0.02	0.13	0.57	0.16
4	0.00	0.03	0.27	0.00	0.13	0.05	0.12	0.07	0.31	0.09	0.10	0.11	0.39	0.21
5	0.00	0.13	0.43	0.02	0.13	0.28	0.25	0.32	0.50	0.50	0.08	0.34	0.30	0.50
6	0.01	0.06	0.25	0.00	0.19	0.50	0.48	0.59	0.29	0.55	0.24	0.59	0.27	0.64
7	0.04	0.01	0.08	0.00	0.01	0.44	0.36	0.57	0.09	0.39	0.30	0.66	0.04	0.54
8	0.06	0.13	0.01	0.00	0.11	0.09	0.15	0.28	0.02	0.13	0.08	0.13	0.03	0.34
9	0.04	0.09	0.01	0.00	0.06	0.17	0.04	0.23	0.01	0.21	0.08	0.20	0.01	0.73
10	0.03	0.03	0.06	0.00	0.00	0.04	0.08	0.09	0.07	0.15	0.20	0.31	0.05	0.17

Table S4. Predicted off-target loci.

The off-target loci were predicted by using CRISPR-GE online website (http://skl.scau.edu.cn).

		Gene	Region	Sequence	off-score
EMB-sgRNA5	*EMB*-sgRNA5	AT2G25660 (*EMB2410*)	3' UTR	TATCCATATGAATCCGACAGagg	/
	off-target 5-1	AT5G05680	CDS	TACCCAAACGAATCCGACAGagg	0.346
	off-target 5-2	AT1G60070 AT1G60072	intergenic	AAACCTTCTGAATCCGACAGagg	0.219
	off-target 5-3	AT2G46560	CDS	TTTCCACAAGAATTAGACAGagg	0.076
EMB-sgRNA8	*EMB*-sgRNA8	AT2G25660 (*EMB2410*)	3' UTR	ATATAAGCCGTTTGTTGCAAagg	/
	off-target 8-1	AT3G63340	CDS	TTATAAACAGTTTGTTGCAAtgg	0.857
	off-target 8-2	AT5G51550 AT5G51560	intergenic	GTTTAACCTGTTTGTTGTAAagg	0.273
	off-target 8-3	AT4G14770 AT4G14780	intergenic	ATATCATTTGTTTGTTGCAAcgg	0.219
EMB-sgRNA9	*EMB*-sgRNA9	AT2G25660 (*EMB2410*)	3' UTR	ATCCGACAGAGGAAGACAAAagg	/
	off-target 9-1	AT5G13740	3' UTR	ATCAGACAAAGCAAAACAAAagg	0.27
	off-target 9-2	AT4G27620	CDS	AACCGACAGAGGAAGATTTAagg	0.106
	off-target 9-3	AT5G18065	intron	ATCCAACAGAGAAAGAAAAAaag	0.098
EMB-sgRNA10	*EMB*-sgRNA10	AT2G25660 (*EMB2410*)	3' UTR	TGCACTTCCTTTGCAACAAAcgg	/
	off-target 10-1	AT5G66680	CDS	TGCACATCGTTAGCAACATAcgg	0.231
	off-target 10-2	AT4G00800	CDS	TGCTCTATCTTTTCAACAAAggg	0.146
	off-target 10-3	AT1G45332	CDS	GGCTCTTCTCTTCCAACAAAcgg	0.12
ROS1-sgRNA2	*ROS1*-sgRNA2	AT2G36490 (*ROS1*)	3' UTR	GCTAACCTCGCCTAATCCGTtgg	/
	off-target 2-1	AT3G15870	intron	CCTAACCCTGACTAAACCGTtgg	0.426
	off-target 2-2	Chr.2	intergenic	CCTAATCTCGCCTAATCTTTtgg	0.284
	off-target 2-3	AT4G34555	CDS	TCTAAACCCGCCAAATCCGGtgg	0.113
	off-target 2-4	AT2G16365	CDS	TCTAAACCCGCCAAATCCGGtgg	0.113

Table S5. Mutation frequencies of predicted off-target sites.

Mutation frequencies were analyzed by PCR followed by sequencing using the T1 transformants.

	single sgRNA		multiple sgRNAs			
	analyzed transformants	mutation frequency	analyzed transformants	mutation frequency	analyzed transformants	mutation frequency
	EMB-GFP-sg5		*EMB-GFP*-sg5555		*EMB-GFP*-sg58910	
off-target5-1	22	1.77%	26	1.37%	23	1.10%
off-target5-2	22	7.53%	26	7.20%	23	9.33%
off-target5-3	22	1.97%	26	2.43%	23	1.80%
	EMB-GFP-sg8				*EMB-GFP*-sg58910	
off-target8-1	46	3.23%			23	2.63%
off-target8-2	46	3.12%			23	2.93%
off-target8-3	46	1.37%			23	2.80%
	EMB-GFP-sg9				*EMB-GFP*-sg58910	
off-target9-1	18	3.33%			23	3.33%
off-target9-2	18	1.17%			23	1.30%
off-target9-3	18	3.30%			23	4.6%
	EMB-GFP-sg10				*EMB-GFP*-sg58910	
off-target10-1	24	1.03%			23	1.83%
off-target10-2	24	4.77%			23	3.4%
off-target10-3	24	3.47%			23	3.97%
	ROS1-GFP-sg2		*ROS1-GFP*-sg222		*ROS1-GFP*-sg2222	
off-target2-1	84	0.90%	96	1.71%	96	1.01%
off-target2-2	84	2.96%	96	2.80%	96	2.68%
off-target2-3	84	0.82%	96	1.74%	96	0.84%
off-target2-4	84	1.36%	96	1.80%	96	1.10%

Table S6. Sequences of primers.

**Primer name**	Primer sequence (5' - 3')
**sgRNA**	
EMB-GFP-sg5	TATCCATATGAATCCGACAGagg
EMB-GFP-sg8	ATATAAGCCGTTTGTTGCAAagg
EMB-GFP-sg9	ATCCGACAGAGGAAGACAAAagg
EMB-GFP-sg10	TGCACTTCCTTTGCAACAAAcgg
**Cloning promoters**	
DD45 promoter	AAATGTTCCTCGCTGACGTAAGAAGAC
	AAATGTTCCTCGCTGACGTAAGAAGAC
***GFP* donor construction**	
GFP	ATGGTGAGCAAGGGCGAGGAG
	TTACTTGTACAGCTCGTCCATGCCGT
EMB2410-GFP-5' arm	GGCATGGCTCATCCAAAATTTATCAAACC
	GTCTTGTGATGTAGCAGAGTATTCAAAAAGCA
EMB2410-GFP-3' arm	CTTCCTCTGTCGGATTCATATGGATATAAGC
	GAGATGTTAAAGGAGCATGAGGAAACGTTAAA
**Genotyping**	
EMB2410-GFP Precise GT	GGACCAGAGCTGAGGATAATG
	GGTCTAGTGATTCTGAAGCC
EMB2410-GFP Total GT	GGACCAGAGCTGAGGATAATG
	CTTGTTGCACCAATCAGTCAATG
Cas9	TGGTGGTGCTCGTCGTATCT
	GACCGGCACAGCATCAAGAA
Bar	TGCACCATCGTCAACCACTAC
	TCAGCAGGTGGGTGTAGAGC
**Potential off-target sites primers**	
EMB2410-sg5-1-OT-F	GTGGCTCTCTTGTTACCTTG
EMB2410-sg5-1-OT-R	TGGAGTTGAACGCTGGTTAG
EMB2410-sg5-2-OT-F	GAATGGATTCATGACCGGAG
EMB2410-sg5-2-OT-R	GATAGCTCCACAACGTCATC
EMB2410-sg5-3-OT-F	TCGTAACTTCATCCTCACGG
EMB2410-sg5-3-OT-R	CCTACAGGTTGCCTCTGATC

**DNA analysis for *ROS1***	
ROS1-5' specific F	AAGCATGAACTCGTAAGTTGGAAA
ROS1-3' specific R	CCTCGAGGATGTTGTTGCAAAT
ROS1-5' external F	AGGCTTGCTTTTGGAAAGGTAC
ROS1-3' external R	CAAACGAAGGGAACAGAGAGTT
ROS1-T7EI-F	ATTACCTCGAAGAACGGTCTATTT
ROS1-T7EI-R	AGTTAAGTGAGATCCTCCACATAT
**RNA analysis for *ROS1***	
ROS1-sgRNA1-qF	TGTTTGCTTGCCAACGGATTagg
ROS1-sgRNA2-qF	GCTAACCTCGCCTAATCCGTtgg
ROS1-sgRNA3-qF	GCTTGCCAACGGATTAGGCGagg
ROS1-sgRNA4-qF	TGTATTTGTTTGCTTGCCAAcgg
ROS1-qR	AAAAAGCACCGACTCGGTGC
AtActin-qF1	CGTTTCGCTTTCCTTAGTGTTAGCT
AtActin-qR1	AGCGAACGGATCTAGAGACTCACCTTG
**off target analysis for *ROS1***	
ROS1-off2-1F	GCTCGTTTTCATCAATGGAATCTG
ROS1-off2-1R	AGTGTATGTGTGTGTATGCACATT
ROS1-off2-2F	CATTCAATCCACATCGAGAAAGC
ROS1-off2-2R	CATAAATTTCGCATGTGCGTGAA
ROS1-off2-3F	AAATTCCCGTCATCAAAAAAAGT
ROS1-off2-3R	ACTACAAACGAGTCGAATCATAC
ROS1-off2-4F	GCAAGTAGCCCTAGTGTTAATCT
ROS1-off2-4R	TAAGGGAAATGAAGACGTCTGTC
