# Supplementary material for: Application of multiple sgRNAs boosts efficiency of CRISPR/Cas9-mediated gene targeting in Arabidopsis
Source: BMC Biol. 2024 Jan 17;22:6. doi: 10.1186/s12915-024-01810-7 (PMC10795408; doi:10.1186/s12915-024-01810-7)
Supplement: Supplementary file 4 — Additional file 4: Fig S4. Original uncropped gel pictures. [file 12915_2024_1810_MOESM4_ESM.pdf]

Figure 3A

Full length

Basta

Cas9

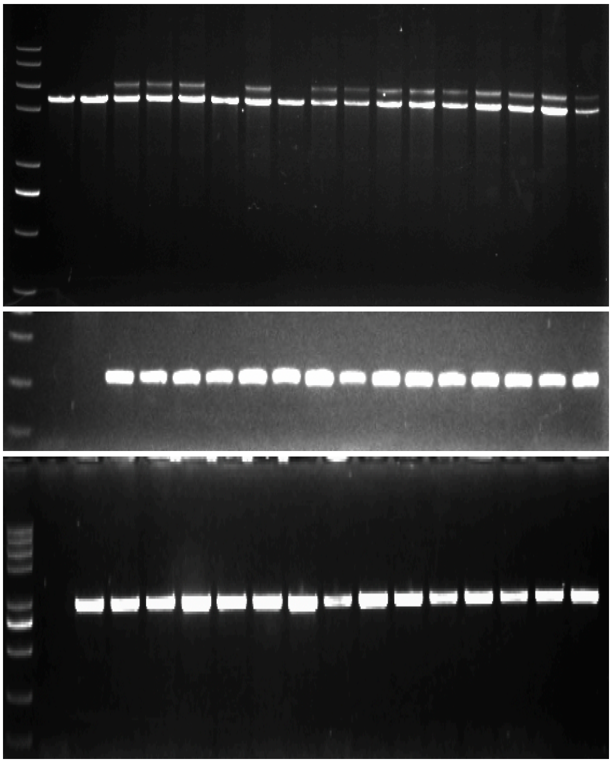

FigS4. Original uncropped gel pictures.

Figure 3B

Full length

5’specific

5’external

3’specific

3’external

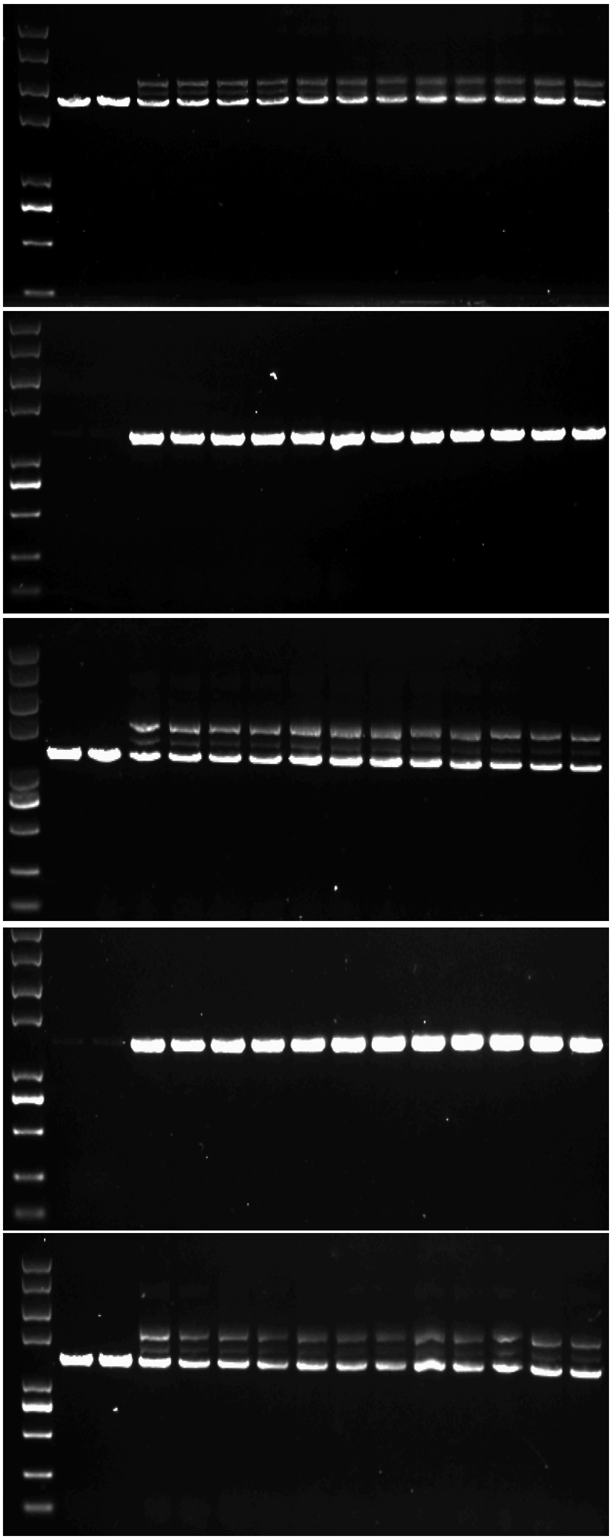

FigS4. Original uncropped gel pictures.

Figure 3C

Full length

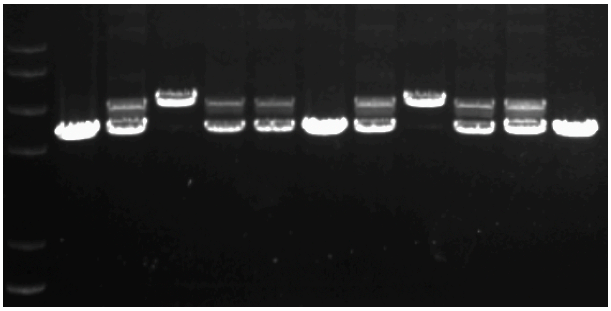

Figure 3D

Full length

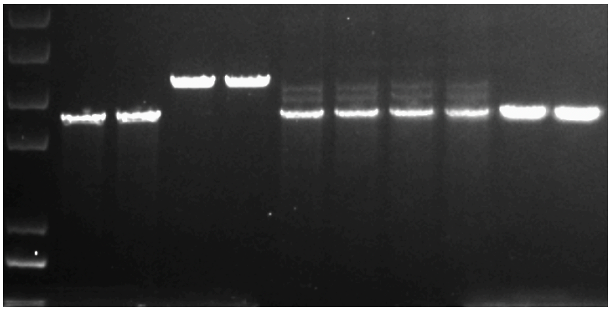

FigS3A

5’ specific

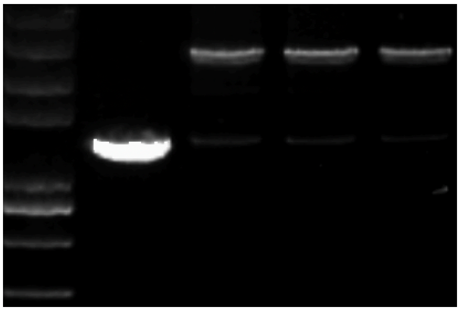

3’ specific

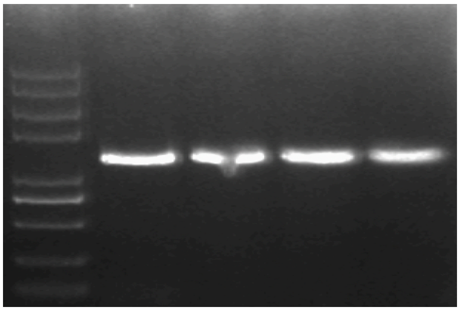

FigS4. Original uncropped gel pictures.
